# Emerging Role of Mechanical Forces in Cell Fate Acquisition

**DOI:** 10.3389/fcell.2022.864522

**Published:** 2022-05-23

**Authors:** Yanina Alvarez, Michael Smutny

**Affiliations:** Centre for Mechanochemical Cell Biology and Division of Biomedical Sciences, Warwick Medical School, University of Warwick, Coventry, United Kingdom

**Keywords:** mechanical forces, cell fate acquisition, morphogenesis, embryonic development, patterning, actomyosin

## Abstract

Mechanical forces are now recognized as key cellular effectors that together with genetic and cellular signals physically shape and pattern tissues and organs during development. Increasing efforts are aimed toward understanding the less explored role of mechanical forces in controlling cell fate decisions in embryonic development. Here we discuss recent examples of how differential forces feedback into cell fate specification and tissue patterning. In particular, we focus on the role of actomyosin-contractile force generation and transduction in affecting tissue morphogenesis and cell fate regulation in the embryo.

## Introduction

A complex interplay between biochemical and physical events on multiple lengths and time scales regulates the formation of tissues and organs during embryonic development. Mechanical forces are now recognized as central players in tissue morphogenesis that drive changes in cell shape, size, proliferation, and movement (Heisenberg and Bellaiche, 2013; Mammoto et al., 2013). These processes rely on dynamic feedback of mechanochemical signals whereby forces are transduced into biochemical signals which in turn control mechanical mechanisms (Hannezo and Heisenberg, 2019; Collinet and Lecuit, 2021). Forces that lead to changes in cell form and function can either be intracellularly generated by contractile actomyosin networks or extrinsically received from the surrounding microenvironment through cell adhesive complexes (cell-cell or cell-extracellular matrix (ECM) receptors) (Lecuit et al., 2011; Heisenberg and Bellaiche, 2013; Mammoto et al., 2013; Vining and Mooney, 2017; Goodwin and Nelson, 2021). Further, cells can also respond to stresses from changes in hydrostatic or hydraulic fluid pressure as observed during early embryonic development (Dumortier et al., 2019; Mosaliganti et al., 2019). Coordination and transmission of mechanical forces allow cells to change shape and position, thereby producing morphogenetic changes at the tissue and organ level.

A crucial event during early embryonic development is the establishment of different cell identities (fates) for specialized function and patterning of tissues and organs. Numerous studies have now established the view that large-scale patterning is achieved by short- or long-range morphogen signaling in tissues in a dose-dependent manner, thereby controlling local activation of transcription factors and modulation of gene expression to determine cell fate (Gilmour et al., 2017). Apart from genetic control of tissue patterning, recent studies highlight a significant role for mechanical forces in cell fate specification, adding another distinct layer of control over cell fate decision making (Mammoto et al., 2011; Brunet et al., 2013; Gordon et al., 2015). Forces generated inside the cell, modulating cell contractility and mechanics (Samarage et al., 2015; Le et al., 2016; Maitre et al., 2016; Mitrossilis et al., 2017) as well as stresses outside the cell such as those produced through hydrostatic pressure can impact on cell fate regulation and tissue patterning (Planas-Paz et al., 2012; Chan et al., 2019). Notably, mechanical signaling through cell–cell and cell–ECM adhesions seems to play a significant role in the interplay between forces and cell fate specification (Martin-Bermudo, 2000; Kuriyama and Mayor, 2009; Maître et al., 2012; Taylor-Weiner et al., 2015; Steed et al., 2016; Barone et al., 2017). Given the multitude of forces present during tissue morphogenesis and the numerous mechanosensitive proteins that can potentially affect cell fate decisions, a major challenge is to delineate which force inputs and which specific effectors are functionally relevant to control cell fate.

In the following chapters, we will briefly discuss the relationship between forces and cell fate and their consequences for tissue and organ development on the basis of recent discoveries in the field with a specific focus on two processes during vertebrate development that serve as excellent model systems of how contractility can control cell fate decisions.

### Feedback Between Cell Fate and Mechanical Forces

The link between forces and cell fate specification is essential for understanding the underlying mechanisms that regulate robust tissue patterning during development (Gilmour et al., 2017). Identifying the mechanical pathways that are responsible for cell fate specification requires quantitative force measurements which are often intricate to accomplish in the embryo. Hence, key findings originate from studies using cultured cells that enable better access and control to investigate the contribution of mechanical signals to changes in cell behavior (Engler et al., 2006; Astudillo, 2020; Petzold and Gentleman, 2021). Such findings revealed, for example, that environmental mechanical cues such as matrix stiffness are key modulators of embryonic stem cell (ESC) differentiation (Engler et al., 2006; McBride and Knothe Tate, 2008; Huebsch et al., 2010; El-Mohri et al., 2017). Furthermore, actomyosin contractility and membrane tension have been shown to guide cell fate and patterning (Fu et al., 2010; Bergert et al., 2021; De Belly et al., 2021), indicating that cortex and membrane tension can actively contribute to cell fate decisions. However, given the precise spatiotemporally controlled biochemical and physical signals together with geometric cues in the embryo, recent studies highlight the need to investigate functional relationships between force and cell fate *in vivo* (Yang et al., 2000; Hove et al., 2003; Desprat et al., 2008; Adamo et al., 2009; Barone et al., 2017).

Notably, mechanical forces controlling cell fate is not a strictly unidirectional pathway. Cell fate can feedback into cytoskeletal tension generation, and this regulatory loop appears to be crucial for robust morphogenesis during development. This typically includes cell–cell adhesion complexes which relay physical signals between cells and are therefore an integral part for integrating mechanosensitive responses at the tissue level. For example, a positive feedback loop between cell–cell contact duration, morphogen signaling, and mesendoderm cell fate specification was observed during early zebrafish gastrulation (Barone et al., 2017). Moreover, compressive forces by the global extension of the germband in *Drosophila* were shown to generate a stretching of the β-catenin-E-cadherin binding site, resulting in the expression of β-catenin target genes including the mesodermal marker *twist* (Desprat et al., 2008). In turn, Twist can control the expression of upstream regulators of actomyosin contractility such as the activation of the Rho-family GTPase RhoGEF2 (Leptin, 1991; Dawes-Hoang et al., 2005; Kolsch et al., 2007; Sandmann et al., 2007). Other known examples of feedback loops between forces and cell fate come from processes regulated by effectors of the Hippo signaling pathway that control organ size during development. Here, the transcriptional co-activator proteins YAP (Yes-associated protein 1) and TAZ (transcriptional coactivator with PDZ-binding motif) are associated with cell proliferation and fate specification and can mechanically be controlled by extracellular matrix rigidity and cell shape (Dupont et al., 2011; Elosegui-Artola et al., 2017). For instance, recent work elegantly demonstrated that cell specification of the micropyle precursor cell (MPC) within the follicular epithelium during zebrafish oogenesis is controlled by nuclear translocation of TAZ (Xia et al., 2019). TAZ triggers massive growth of the MPC, which leads to mechanical compression and deformation of its neighboring cells and, consequently, the depletion of nuclear TAZ in these cells. This lateral inhibition mechanism triggers a positive feedback loop, facilitating TAZ-dependent growth of the dominant cell while at the same time limiting growth in the surrounding cells (Xia et al., 2019).

In the next chapters, we will discuss recent findings on how actomyosin anisotropies can lead to different cell fates during embryogenesis with a particular focus on early heart development in zebrafish and first lineage segregation in the mouse.

### Trabeculation During Zebrafish Heart Development

**FIGURE 1:**
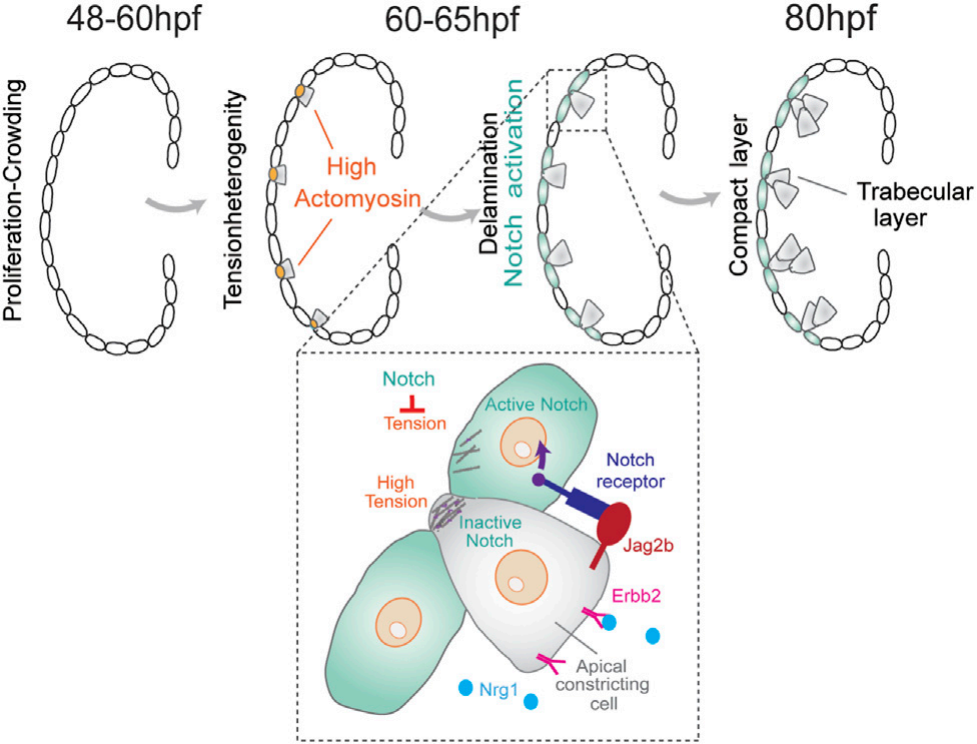
During cardiac trabeculation in zebrafish (between 60 and 65 hpf), proliferation-induced crowding leads to tension heterogeneity in cardiomyocytes. CMs with higher tension constrict their apical domain and delaminate to seed the trabecular layer. This delamination triggers activation of Notch signaling in adjacent compact layer CMs, thereby establishing a distinct CM fate for these two layers. The coordination between Notch and Erbb2 pathways between neighboring cells produces a distinctive pattern of cell shape and fate for the trabecular and compact layer formation.

Heart development in vertebrates undergoes complex morphogenetic transformations during cardiac trabeculation, a process where sheet-like muscular structures form as a result of cardiomyocytes' extrusion and expansion into the lumen of the ventricular chambers (Staudt and Stainier, 2012). Although the zebrafish heart has only two chambers instead of four as the mammalian counterpart, the major components are conserved and similar cellular and molecular pathways are implicated during heart development (Moorman and Christoffels, 2003). In zebrafish, the myocardium transforms from a monolayer at 48 h post-fertilization (hpf) to a complex three-dimensional (3D) structure that consists of two cell types: the outer compact layer (CL) cardiomyocytes encircling the inner trabecular layer (TL) cardiomyocytes (Figure 1). The Notch signaling pathway has been reported to play an important role in fate specification during trabecular morphogenesis (Samsa et al., 2015). A zebrafish line with a Notch reporter from the Epstein–Barr virus terminal protein 1 (TP1) gene was utilized to study cell fate specification during trabecular morphogenesis. Notch reporter TP1 was shown to be activated in CL cardiomyocytes but not in TL cardiomyocytes (Han et al., 2016; Jimenez-Amilburu et al., 2016). Moreover, abrogating myocardial Notch led to ectopic trabeculation (Han et al., 2016). In mouse embryos, however, Notch signaling activation is essential for ventricular trabeculation initiation, but the inactivation of myocardial Notch does not affect heart development (Grego-Bessa et al., 2007; Salguero-Jimenez et al., 2018), which points to differences in Notch-dependent regulation of heart development across species. In zebrafish, differential myocardial fate requires binding of epidermal growth factor neuregulin 1 (Ngr1) to Erb-B2 receptor tyrosine kinase 2 (Erbb2) which leads to its phosphorylation and downstream signaling (Han et al., 2016). Endocardial Nrg1 activates myocardial Erbb2 signaling, which triggers the expression of the Notch receptor ligand, Jag2b. In turn, Jag2b activates Notch signaling in neighboring cardiomyocytes, which inhibits Erbb2 expression. This regulatory feedback mechanism prevents excessive cell internalization of the embryonic outer cell layer to generate a distinctive morphology and fate during early heart development (Figure 1).

A recent study discovered that cardiomyocytes with higher contractility delaminate even in the absence of the Nrg–Erbb2 pathway (Priya et al., 2020). In this model, tissue crowding induces local differences in cell shape and tension to initiate cardiomyocytes with higher contractility to segregate by apical constriction. Moreover, changes in actomyosin contractility were shown to be sufficient to trigger differential apicobasal polarity and fate (Priya et al., 2020). This hypothesis is based on the fact that myocardial Notch reporter expression correlates with the apical surface area of cardiomyocytes. Apical domain length quantifications showed that cells with higher expression levels of TP1 in the CL layer have larger apical domains than those in delaminating cardiomyocytes (Priya et al., 2020). Lastly, myocardial wall patterning was postulated to rely on a Notch signaling feedback pathway. In particular, Notch signaling is activated in neighboring CL cardiomyocytes which suppresses the actomyosin machinery in these cells and limits excessive delamination (Figure 1). The mechanism for the Notch-mediated lateral inhibition is still unknown, but a model considering contact area dependence predicts that smaller cells are more likely to be selected by the lateral inhibition process than larger cells (Shaya and Sprinzak, 2011).

Although major advances in understanding heart morphogenesis have been achieved, high-resolution 3D imaging of beating hearts during developmental stages remains challenging. Recent advances in live imaging of a developing mouse heart coupled with computational segmentation accomplished precise tracking of cell fate decisions during embryonic development (Yue et al., 2020). This is a crucial first step in modeling heart morphogenesis at a single-cell resolution in order to enhance our understanding of heart development.

### First Lineage Segregation in Mouse Embryos

**FIGURE 2:**
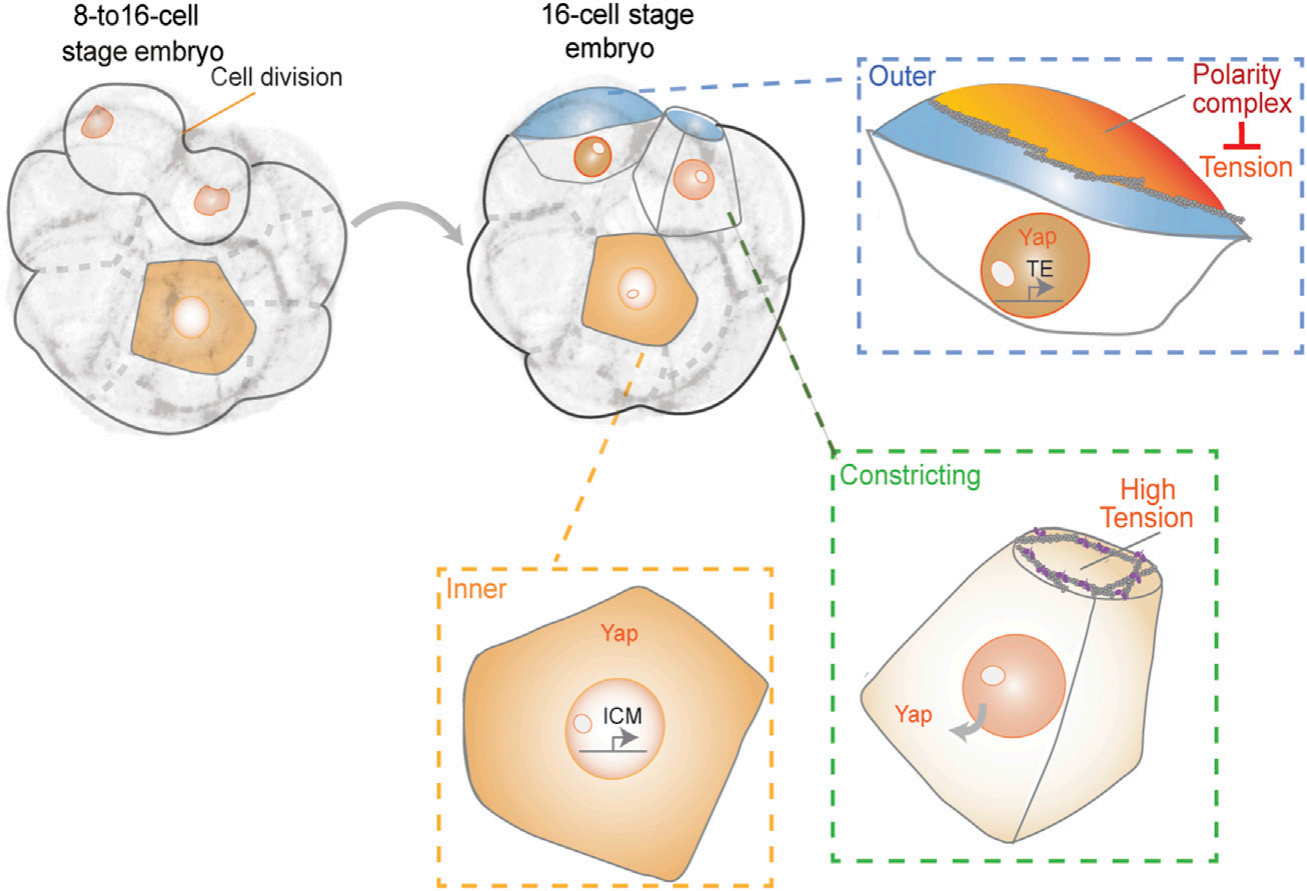
First lineage segregation during 8- to 16-cell transition. The resulting daughter cells show differences in polarity, contractility, and exposed surface area. Together, these properties may control cell fate acquisition, resulting in appropriate partitioning of ICM and TE cells during patterning of the blastocyst.

During the preimplantation stages of mammalian embryonic development, cells of the embryo physically segregate into the pluripotent inner cell mass (ICM), which contains the precursors for all cells in the body, and the outer trophectoderm (TE) layer that will form the placenta (White et al., 2018). In the mouse embryo, this lineage segregation starts after the 8-cell stage. It has been suggested that asymmetric cell divisions are the main mechanism to ensure ICM formation (Yamanaka et al., 2006; Zernicka-Goetz et al., 2009). However, asymmetric divisions are infrequent, and the first inner cells originate primarily from cell internalization events. During this process, blastomeres divide with tilted angles, and one daughter internalizes gradually via cortical tension-dependent apical constriction (Samarage et al., 2015) (Figure 2). Apicobasal polarity and Hippo signaling are believed to be the key molecular mechanisms by which outer and inner cells control their fate (Plusa et al., 2005; Sasaki, 2017; White et al., 2018). The establishment of apical polarity by Par-aPKC components in the outer (polar) cells was shown to promote the nuclear localization of YAP, which upregulates the expression of Cdx2, a transcription factor essential for TE-fate maturation (Strumpf et al., 2005; Ralston and Rossant, 2008). In contrast, inner (apolar) cells lack apical polarity and YAP remains cytoplasmic through phosphorylation by the Hippo signaling pathway component Lats. Cytoplasmic YAP fails to activate homeobox transcription factor Cdx2 expression to promote a pluripotent fate (Nishioka et al., 2009; Sasaki 2017). Yet, it is unclear when YAP and Cdx2 start to be differentially regulated during inner-outer segregation (Hirate et al., 2015; Sasaki 2017). Recent reports indicate that the F-actin-rich apical domain might be asymmetrically inherited during cell division to differentially control YAP and Cdx2 (Maitre et al., 2016; Korotkevich et al., 2017). According to this model, segregation of the apical domain generates both polarized and unpolarized blastomeres, which are defined by the different levels of apical aPKC and myosin 2. Unpolarized cells showed higher cortical levels of myosin 2 than polarized ones, and the differences in contractility determined their sorting into inner and outer positions (Maitre et al., 2016). Polar daughter cells that inherited the apical domain displayed lower contractility and remained in the outer position whereas apolar cells internalized.

Furthermore, by using a reduced system in which two blastomeres are isolated from a 16-cell stage embryo, it was shown that the apical domain recruits a spindle pole to ensure its differential distribution upon division (Korotkevich et al., 2017). According to this model, the inheritance of the apical domain is sufficient for the daughter cell to adopt TE fate. In contrast to this model, the apical domain seems to disassemble when blastomeres divide before being re-established *de novo* after cytokinesis (Zenker et al., 2018). These results demonstrate that polarity establishment does not occur immediately after division. In agreement with these observations, it was recently reported that keratins form long-lived filaments that become asymmetrically retained by outer daughter cells. Keratin filaments may stabilize the cortex to promote the subsequent establishment of apical Par-aPKC components (Lim et al., 2020). Despite direct links between the Hippo pathway and F-actin (Leung and Zernicka-Goetz 2013; Sasaki 2017), the direct role of actomyosin-generated tension in controlling cell fate during early mouse embryonic development remains unclear. Yet, the observation that cortical contractility causes blastomeres to become inner cell-like with respect to phosphorylated YAP localization and Cdx2 levels and independent of their external position, favors such an idea (Maitre et al., 2016). These results suggest the possibility that YAP may sense cortical tension independently of apical polarity. Moreover, in a recent work, a correlation between levels of nuclear YAP and the proportion of the exposed apical surface area of each blastomere at the 16-cell stage was observed (Royer et al., 2020). This suggests that cells may sense the proportion of their surface area exposed and signal to the nucleus by modulating the subcellular localization of YAP. The authors suggested a possible feedback loop between apical cell surface area and YAP localization. Certain cells that exhibited a lower proportion of exposed surface area after cell divisions from 8- to 16-cell stage, displayed lower nuclear YAP levels and subsequently internalized (Royer et al., 2020). However, the precise underlying mechanisms of regulation remain unclear and future studies will be needed to gain a complete understanding of this process.

## Discussion

Understanding the crosstalk between cell- and tissue-scale mechanics and cell fate specification is essential to uncover the key mechanisms that regulate robust tissue patterning during development. Mechanical forces are now recognized as essential control mechanisms for tissue integrity and function by regulating cellular processes such as tension, polarity, and adhesion during development. In this mini-review, we revisited recent studies that illustrate the impact of forces on cell-fate specification during embryonic development with a particular focus on zebrafish heart development and first lineage segregation in the mouse. Notably, the establishment of force anisotropies seems to be a conserved feature in both systems to drive changes in cell identities and suggests that local differences in cell shape and contractility might be a more general mechanism in mechanical regulation of cell fate across various species. In this regard, it will also be critical to identify mechanosensitive proteins and their specific contribution to cell fate changes such as mechanosensitive ion channels at the plasma membrane including TRP (Liu and Montell, 2015) and Piezo1 (Ridone et al., 2019), or mechanoresponsive proteins at cell adhesion sites such as α-catenin and vinculin.

Moreover, recent work revealed that mechanical forces also impact nuclear morphology and processes within the nucleus (Kirby and Lammerding, 2018; Lomakin et al., 2020; Venturini et al., 2020). Nuclear responses to mechanical force include adaptations in chromatin architecture and transcriptional activity that trigger changes in cell state (Hampoelz and Lecuit, 2011). These force-driven changes also influence the mechanical properties of chromatin and nuclei themselves to prevent aberrant alterations in nuclear shape and maintain genome integrity (Uhler and Shivashankar 2017). Linking cell and nuclear mechanics to events directly controlling gene expression involved in cell-fate specification will be an important endeavor for future studies to completely understand developmental programs.
